# *DBI* as a Novel Immunotherapeutic Candidate in Colorectal Cancer: Dissecting Genetic Risk and the Immune Landscape via GWAS, eQTL, and pQTL

**DOI:** 10.3390/biomedicines13051115

**Published:** 2025-05-04

**Authors:** Ting Tian, Huan Han, Jingtao Huang, Jun’e Ma, Ruoxi Ran

**Affiliations:** 1 Department of Clinical Laboratory, Maternal and Child Health Hospital of Hubei Province, Tongji Medical College, Huazhong University of Science and Technology, Wuhan 430070, China; 13554078668@163.com (T.T.); ma18893718032@163.com (J.M.); 2 Department of Clinical Laboratory, Institute of Translational Medicine, Renmin Hospital of Wuhan University, Wuhan 430060, Hubei, China; hh940622@whu.edu.cn (H.H.); jthuang@whu.edu.cn (J.H.)

**Keywords:** colorectal cancer, drug target, DBI, Mendelian randomization, tumor microenvironment

## Abstract

**Background:** Colorectal cancer (CRC) is a leading cause of cancer-related mortality worldwide. Identifying drug targets associated with CRC is crucial for developing targeted therapies. **Methods:** MR (IVW, Wald ratio, weighted median, and MR-Egger) and SMR analyses were used to screen candidate genes associated with CRC risk. Further validation was performed using The Cancer Genome Atlas (TCGA) to assess gene expression patterns and prognostic significance. Additionally, immune infiltration analysis was conducted to characterize the tumor immune microenvironment. Drug prediction was performed to explore potential therapeutic interventions. **Results:** Eight genes were identified associated with CRC. *IGFBP3*, *CD72*, *SERPINH1*, *CHRDL2*, *LRP11*, and *SPARCL1* were linked to an increased risk of CRC, whereas *DBI* and *HYAL1* were associated with a decreased risk of CRC. Notably, DBI exhibited a potentially favorable immune profile, negatively correlated with Tregs and MDSCs while positively associated with activated CD8^+^ and CD4^+^ T cells. **Conclusions:** Eight genes were identified as associated with CRC, among which *DBI* exhibited a potential protective role, correlating with improved patient survival, enhanced immune activation, and increased responsiveness to immunotherapy. The remaining proteins demonstrated diverse and complex functions within the tumor immune microenvironment, providing novel insights for the development of precision diagnostics and immunotherapeutic strategies.

## 1. Introduction

Colorectal cancer (CRC) is the most commonly diagnosed cancer of the digestive tract. In 2020, approximately 1.9 million people were diagnosed with CRC, with an estimated 935,000 deaths [1]. Despite significant advancements in early detection and therapeutic strategies, the prognosis of patients with advanced or metastatic CRC remains poor: five-year survival is under 14% [2]. Current treatments include surgery, chemotherapy, targeted agents, and immunotherapy. While surgical resection can be curative in early stages, advanced or metastatic CRC continues to carry a dismal prognosis. Chemotherapy, the cornerstone of systemic treatment for CRC, is frequently limited by substantial toxicities and suboptimal response, particularly in patients with metastatic disease. Targeted therapies, such as anti-epidermal growth factor receptor (anti-EGFR) and anti-vascular endothelial growth factor (anti-VEGF) monoclonal antibodies, have shown promise in patients with KRAS/NRAS wild-type tumors and microsatellite instability-high (MSI-H) cancers. However, mutations in the KRAS and BRAF genes have substantially diminished the efficacy of these therapies. Furthermore, despite promising results from early-phase trials of immune checkpoint inhibitors in MSI-H and mismatch repair-deficient tumors, additional studies are required to confirm their safety and efficacy [3,4,5,6]. The current reliance on single-agent regimens or narrow combinations underscores the need for more innovative therapeutic strategies for CRC.

In addition, the inherent molecular heterogeneity of CRC and the tumor microenvironment (TME) further complicate treatment strategies [7]. The TME is a complex network of cellular and acellular components—cancer cells, immune cells, extracellular matrix (ECM), cytokines, and signaling molecules—that together drive tumor progression, immune evasion, and therapy resistance. TME–tumor interactions have been shown to alter plasma cytokine levels and the proteome, potentially promoting tumor progression and metastasis [8,9,10]. Notably, immune checkpoints, such as the programmed cell death 1 (PD-1) and the cytotoxic T cell-associated protein 4 (CTLA-4), have emerged as central regulators of anti-tumor immunity. Studies have shown that the PD-1 receptor inhibitor nivolumab and the CTLA-4 inhibitor ipilimumab have demonstrated significant efficacy in both early-stage and advanced CRC [11,12]. Pelka et al. [13] constructed a high-resolution single-cell atlas of the CRC TME, uncovering diverse immune and stromal populations, which included regulatory T cells (Tregs), myeloid-derived suppressor cells (MDSCs), and tumor-associated fibroblasts. These findings highlight their potential as therapeutic targets. Moreover, the study emphasized the importance of metabolic reprogramming within the TME as a critical regulator of immune cell function. Collectively, these results underscore the tumor–immune–metabolic axis as a promising avenue for therapeutic intervention. Targeting immunosuppressive cells and metabolic pathways within the TME—particularly in combination with current treatment strategies—may enhance therapeutic efficacy in CRC.

In recent years, Mendelian randomization (MR) has emerged as a powerful tool for inferring causal relationships between genetic variants and disease outcomes [14]. MR utilizes genetic variants as instrumental variables to overcome the limitations of observational studies, such as confounding and reverse causality. By leveraging genetic variants linked to modifiable exposures, such as gene expression or protein levels, MR can evaluate the causal impact of these factors on CRC risk [15]. This method helps pinpoint genetic factors that not only contribute to disease susceptibility but also serve as potential therapeutic targets. A recent MR study revealed that several genetically predicted immune cell subsets—including B cells, CD8^+^ T cells, Tregs, and monocytes—may play causal roles in CRC development [16]. These findings highlight the importance of immune cell composition in CRC pathogenesis and suggest that modulating the abundance or functional state of key immune populations could provide novel therapeutic opportunities. Reprogramming immune cell states within the tumor microenvironment may help overcome immunosuppression and enhance anti-tumor immunity in CRC.

Genome-Wide Association Studies (GWASs) have been instrumental in identifying common genetic variants associated with CRC risk. With the increasing availability of large, well-powered datasets, GWASs have uncovered numerous loci that influence CRC susceptibility [17]. Recent multi-omic analyses have greatly deepened our understanding of CRC genetics. In a pivotal study, Law et al. integrated genomic and transcriptomic data from over 100,000 CRC cases and 150,000 controls across European and East Asian populations, identifying 205 risk loci. These loci are enriched in pathways vital to tumorigenesis, including immune regulation, epithelial homeostasis, lipid metabolism, WNT signaling, and TGF-β signaling [18]. Building on these insights, growing attention is being directed toward the tumor–immune–metabolic interface, where immunosuppressive pathways and metabolic constraints converge to shape disease progression and treatment resistance. However, despite the substantial insights gained from GWAS, translating these genetic associations into actionable therapeutic targets remains a considerable challenge. The integration of GWAS data with functional genomics approaches, such as expression quantitative trait loci (eQTL) and protein quantitative trait loci (pQTL) analyses, can bridge this gap by identifying how genetic variants affect gene expression and protein production. EQTLs reveal genetic variants that influence gene expression levels, potentially uncovering key regulatory mechanisms involved in CRC development. Likewise, pQTLs identify genetic variants that regulate protein abundance, offering insights into the functional consequences of genetic variation on disease progression [19,20]. Integrating MR with eQTL and pQTL data provides a robust framework to reveal how genetic variants drive molecular changes that affect disease risk [21,22]. These integrative approaches allow for the identification of novel drug targets and biomarkers that may not have been apparent through traditional GWAS or gene expression studies alone.

In this study, we utilized GWAS, eQTL, and pQTL data to dissect the genetic architecture of CRC and pinpoint candidate therapeutic targets. Leveraging large-scale resources (UK Biobank, The Cancer Genome Atlas (TCGA), and other consortia), we performed comprehensive analyses to uncover variants that modulate gene expression and protein levels, and evaluated their effects on CRC susceptibility. We aimed to delineate causal pathways that could inform novel CRC therapeutics and discover biomarkers predictive of prognosis and therapeutic response.

## 2. Materials and Methods

### 2.1. Research Design

**Figure 1:**
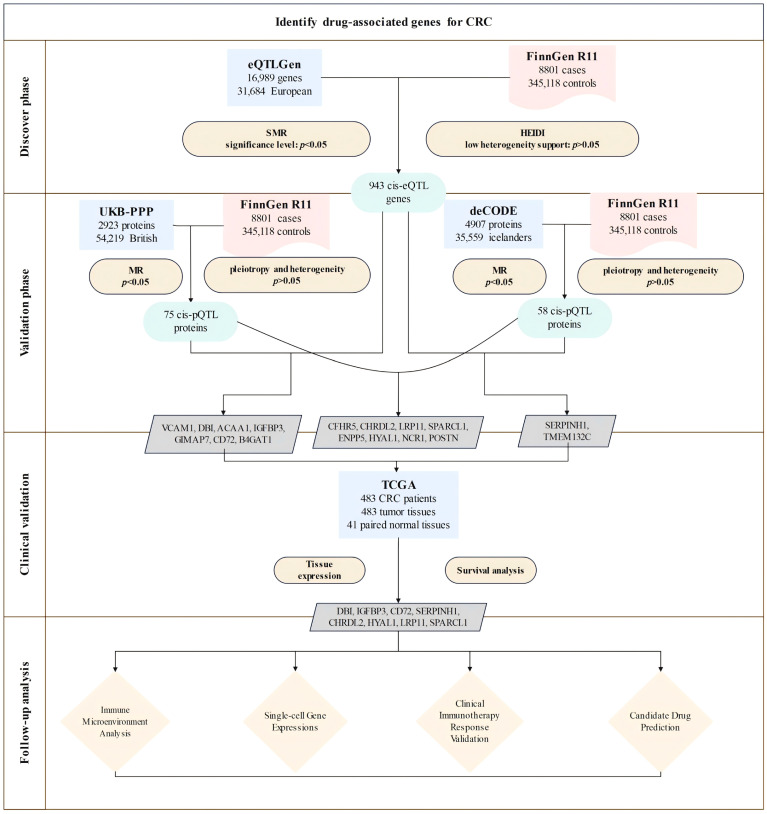
Overview of the study design. The study involved four phases: (1) discovery, integrating eQTLGen and FinnGen data via SMR and HEIDI tests, identifying 943 CRC-associated cis-eQTL genes; (2) validation through pQTL analysis using UKB-PPP and deCODE datasets, which identified proteins significantly associated with CRC risk; (3) clinical validation using expression and survival analyses in TCGA, selecting eight prioritized candidates (*DBI*, *IGFBP3*, *CD72*, *SERPINH1*, *CHRDL2*, *HYAL1*, *LRP11*, *SPARCL1*); (4) follow-up analyses including immune microenvironment profiling, single-cell expression analysis, clinical immunotherapy response validation, and candidate drug prediction.

In this study, we established an integrated analytical framework (Figure 1) to systematically identify and validate druggable genes in CRC. We first applied instrumental variables by utilizing cis-eQTL data from the eQTLGen Consortium. We then conducted summary-based Mendelian randomization (SMR) analysis along with heterogeneity in dependent instruments (HEIDI) tests to identify genes with potential causal effects on CRC.

Next, we validated these associations at the protein level using cis-pQTL data from the UK Biobank Proteome Plasma Proteins (UKB-PPP) and the deCODE datasets, accompanied by sensitivity analyses to confirm result robustness.

We then assessed clinical relevance using TCGA data to evaluate gene expression patterns and their prognostic significance. Additionally, we integrated immune cell infiltration and immune checkpoint analyses to comprehensively characterize the tumor immune microenvironment. Furthermore, functional enrichment analyses were employed to investigate potential mechanisms and pathways underlying disease pathogenesis. DSigDB-based drug prediction was utilized to explore candidate targeted drugs. Collectively, these multifaceted approaches aim to support precision treatment strategies in CRC.

### 2.2. Exposure Data

We obtained eQTL data from the eQTLGen Consortium (https://eqtlgen.org/, accessed on 27 January 2025) [23], which encompasses 16,989 cis-eQTL genes derived from 31,684 blood samples of individuals with European ancestry. To elucidate the genetic underpinnings of plasma protein concentrations, we examined two large-scale GWAS datasets: the UKB-PPP and the deCODE Health study. In the UKB-PPP dataset, protein levels for 2923 proteins were quantified using the Olink platform in 54,219 UK Biobank participants [24], enabling us to interpret the influence of genetic variants. In parallel, the deCODE Health study employed a high-throughput SomaScan platform to assess 4907 aptamer–protein pairs in 35,559 Icelandic subjects [25].

For our instrumental variable analysis, we exclusively selected index cis-pQTLs that achieved genome-wide significance (*p* < 5 × 10^−8^) and were located within 1 Mb of their respective protein-coding genes. These candidate instruments were validated using linkage disequilibrium data from European individuals in the 1000 Genomes Project.

### 2.3. Outcome Data

We accessed the CRC cohort dataset from FinnGen Release 11, which was published in June 2024 (https://www.finngen.fi/, accessed on 27 January 2025) [26]. This dataset includes 8801 confirmed CRC cases and 345,118 individuals classified as healthy.

### 2.4. SMR Analysis and HEIDI Test

By integrating data from GWAS and eQTL studies, the SMR and HEIDI methods help determine whether observed associations stem from pleiotropy or genetic linkage [27]. The SMR approach employs summary-level data to explore the relationship between particular traits and gene expression levels, considering a *p*-value below 0.05 as indicative of a significant association. In contrast, the HEIDI test examines whether the association detected by SMR is driven by a single genetic variant affecting multiple traits (pleiotropy) or by two distinct yet closely located variants (linkage). A HEIDI *p*-value under 0.05 supports a pleiotropic effect [28].

SMR is a specialized form of MR that integrates GWAS and eQTL summary data to test whether the effect of a single top cis-eQTL variant on gene expression is pleiotropically associated with a complex trait, using the HEIDI test to distinguish true pleiotropy from linkage disequilibrium. Together, these complementary analyses provide robust evidence for a causal link between CRC and gene expression, as revealed by the SMR findings.

### 2.5. Mendelian Randomization Analysis

The MR analysis in this study was conducted using the “TwoSampleMR” R package (version 0.5.7). Several quality control procedures were applied to ensure the selection of reliable and robust instrumental variables (IVs). First, we retained only variants with an F-statistic of ≥10 (F = (beta/se)^2^). Then, we focused on variants with low linkage disequilibrium (LD; r^2^ < 0.1), as confirmed using the European reference panel from the 1000 Genomes Project, to ensure their independence [29]. Lastly, Steiger filtering was applied to eliminate genes where the genetic instruments explained more of the variance in the outcome than in the exposure, addressing potential reverse causation concerns [30].

For genes with multiple IVs in the primary analysis, several methods were used to aggregate SNP estimates: Inverse-Variance Weighted (IVW), Wald ratios, weighted median, and MR-Egger. The IVW method, which assumes that all instruments are valid, was employed for the meta-analysis of SNP estimates, as it offers superior statistical power under the no-pleiotropy assumption [31]. The weighted median method provides a reliable causal estimate even if up to 50% of the IVs are invalid [32]. Meanwhile, the MR-Egger method estimates the causal effect by calculating the slope of the Egger regression and accounts for pleiotropy by including an intercept that reflects the average influence of pleiotropic SNPs on the outcome [33].

Additionally, statistical tests were performed to further assess horizontal pleiotropy and heterogeneity. Cochran’s Q test was used to evaluate heterogeneity among the genetic instrument estimates, as significant heterogeneity may indicate pleiotropy or differing effects of the IVs on the outcome. To address horizontal pleiotropy, we applied the MR-Egger regression intercept and the MR-PRESSO method to detect and correct for potential directional pleiotropy among the genetic instruments [33,34].

### 2.6. Transcriptomic Profiling and Prognostic Assessment

Gene expression data and corresponding clinical information for CRC were obtained from the TCGA database (https://portal.gdc.cancer.gov, accessed on 27 January 2025). Using R, we downloaded the data in both count and TPM (transcripts per million, a unit of gene expression quantification) formats. The TPM values were normalized with a log2(TPM + 1) transformation. After filtering the samples to include only those with both RNA-sequencing data and clinical information, we selected a total of 524 samples for further analysis.

To assess gene expression levels, we applied the Kruskal–Wallis (KW) non-parametric test to the TPM data. Survival differences between groups were evaluated using Kaplan–Meier (KM) survival analysis and the log-rank test. For the Kaplan–Meier curves, *p*-values and hazard ratios (HRs) with 95% confidence intervals (CIs) were calculated from the log-rank test and univariate Cox regression analysis. All statistical analyses were performed in R software, with results considered statistically significant if the *p*-value was less than 0.05.

### 2.7. Immune Microenvironment Analysis

To further investigate the role of candidate genes in the tumor immune microenvironment (TIME), we utilized TPM data from TCGA and applied the single sample gene set enrichment analysis (ssGSEA) method using the “IOBR” R package (version 0.99) [35]. This analysis examined the correlation between the candidate genes and the infiltration of 28 different immune cell types, include cell types related to adaptive immunity: activated T cells, central memory (Tcm), effector memory (Tem) CD4^+^ and CD8^+^ T cells, gamma delta T (Tγδ) cells, T helper 1 (Th1) cells, Th2 cells, Th17 cells, regulatory T cells (Treg), follicular helper T cells (Tfh), activated, immature, and memory B cells, as well as cell types related to innate immunity, such as macrophages, monocytes, mast cells, eosinophils, neutrophils, activated, plasmacytoid, and immature dendritic cells (DCs), NK cells, natural killer T (NKT) cells, and MDSCs [36]. Bonferroni correction was applied for multiple comparisons in the ssGSEA analysis involving 8 genes, and a threshold of *p* < 0.00625 (0.05/8) was considered statistically significant.

### 2.8. Analysis of CRC Single-Cell Transcriptomes

Gene expression in CRC was analyzed using single-cell RNA-sequencing (scRNA-seq) data from the GEO database (GSE108989, GSE136394, GSE139555, GSE146771, EMTAB8107, and GSE166555) [37,38,39,40,41,42]. For the single-cell analysis, we obtained scRNA-seq data in HDF5 format (.h5 file extension) and corresponding annotation files from the TISCH2 database (http://tisch.comp-genomics.org/, accessed on 27 January 2025). Data preprocessing was performed using the R packages MAESTRO and Seurat, which included quality control, normalization, scaling, and clustering. Cell types were re-clustered by the t-distributed stochastic neighbor embedding (t-SNE) algorithm, and their identities were validated using standard marker genes. We compared gene expression profiles across various cell types—including tumor, stromal, and immune cells—and visualized the results with heatmaps. The expression value was normalized via the global-scaling normalization method in Seurat to scale the raw counts (UMI) in each cell to 10,000 and then log-transformed.

### 2.9. Clinical Drug Resistance Analysis

Clinical transcriptome analysis of cancer treatment response was conducted using the Cancer Treatment Response gene signature database (CTR-DB, http://ctrdb.ncpsb.org.cn, accessed on 5 February 2025) to assess candidate genes in CRC immunotherapy using GSE154538 [43]. This dataset comprises advanced CRC samples with high TMB (tumor mutational burden), specifically designed to evaluate the efficacy of anti-PD-1 therapy (nivolumab).

### 2.10. Drug Target Prediction

Potential drugs targeting DBI were identified using two drug target prediction platforms: DrugBank (https://go.drugbank.com/, accessed on 5 February 2025) and the Therapeutic Target Database (TTD, https://db.idrblab.net/ttd/, accessed on 5 February 2025). These resources provided comprehensive details, including approved drugs, agents in clinical trials, and experimental compounds, along with their drug IDs, mechanisms of action, and current development stages.

Meanwhile, we submitted DBI to the Drug Signatures Database (DSigDB, https://dsigdb.tanlab.org/DSigDBv1.0/, accessed on 5 February 2025) to identify protein–drug interactions. DSigDB contains 22,527 gene sets and 17,389 compounds linked to 19,531 genes, aiding the discovery of connections between drugs, chemicals, and target genes [44]. DBI was also uploaded to the Enrichr tool (https://maayanlab.cloud/Enrichr/, accessed on 5 February 2025) to predict potential drug candidates targeting this protein [45].

## 3. Results

### 3.1. Discovery of Potential Cis-eQTL Genes and CRC

**Figure 2:**
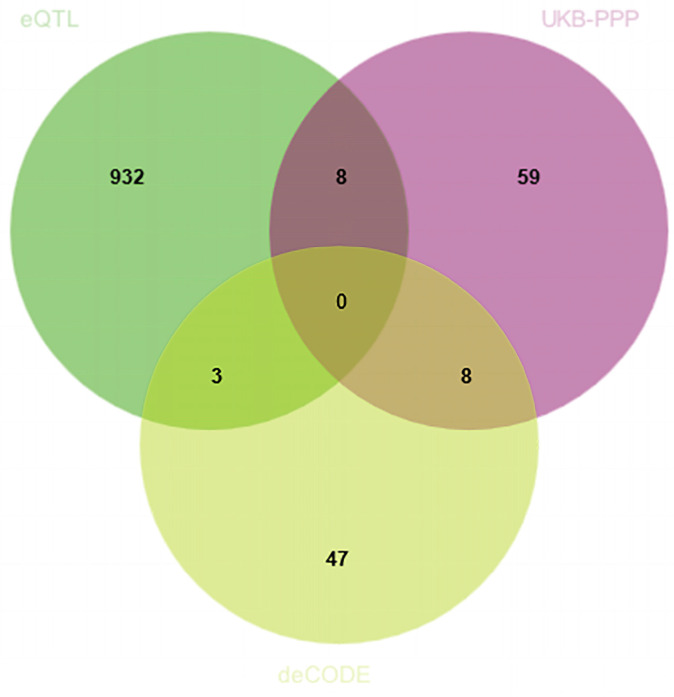
Venn diagram illustrating the overlap of positively identified genes across the eQTLGen, UKB-PPP, and deCODE datasets. Each number represents the count of genes found exclusively in one dataset or shared among two or three datasets.

By integrating eQTLGen, UKB-PPP, and deCODE data, we identified genes significant at both the transcriptomic and proteomic levels. SMR analyses and HEIDI tests were conducted in a discovery cohort of 8801 CRC cases and 345,118 controls from FinnGen. Table S1 details the cis-eQTL variants used, among which 943 showed significant associations with CRC risk (Figure 2). Associations fulfilling HEIDI (*p* > 0.05) and SMR (*p* < 0.05) criteria are listed in Table S1.

### 3.2. MR Analysis of pQTLs in Validation Phase

To further investigate the causal effects of candidate genes on CRC at the protein level, we utilized cis-pQTL protein data from the UKB-PPP and deCODE databases. Due to the absence of corresponding genes, only 11 genes identified during the discovery phase were included in the MR analysis for validation using the cis-pQTL protein data from UKB-PPP and deCODE databases (Figure 2).

In the UKB-PPP dataset, cis-pQTL analysis confirmed significant CRC associations for VCAM1, DBI, ACAA1, IGFBP3, GIMAP7, CD72, and B4GAT1. The results indicated that VCAM1, DBI, ACAA1, GIMAP7, and B4GAT1 were linked to a decreased risk of CRC (Figure 2). These seven proteins were consistent with the SMR results in terms of the direction of effect, and no heterogeneity or horizontal pleiotropy was detected (Table S2). All seven proteins exhibited a consistent direction of impact across the four methods (Figure 2). Steiger filtering further validated the directional relationship between gene expression and disease state (Table S3). HEG1 was excluded due to inconsistent effect direction between discovery and validation.

**Figure 3:**
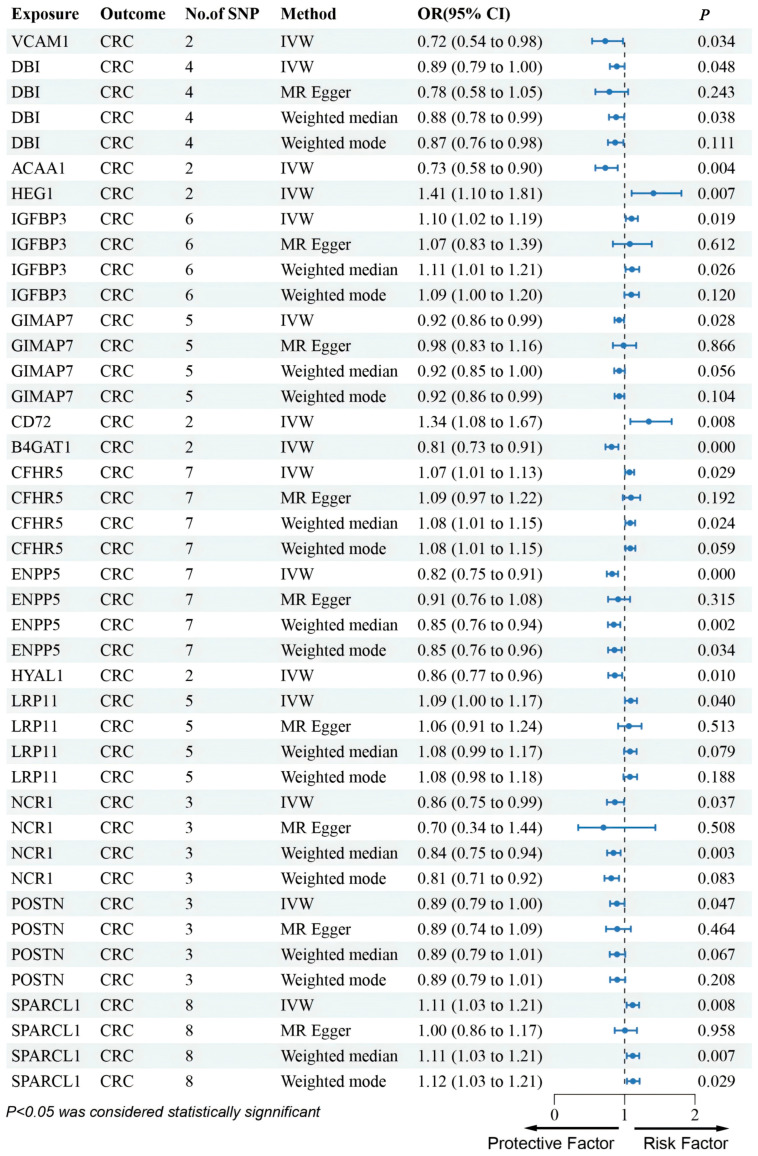
Forest plots illustrating the results of the validation stage for essential cis-pQTLs in UKB-PPP.

In addition, results from the deCODE database revealed that an increased risk of CRC was associated with SERPINH1 and TMEM132C (SERPINH1: OR (95% CI) = 1.47 (1.17–1.85), *p* = 1.01 × 10^−3^; TMEM132C: OR (95% CI) = 1.10 (1.00–1.21), *p* = 4.64 × 10^−2^) (Figure 3). These two proteins were also in agreement with the SMR results regarding the direction of effect, and no heterogeneity or horizontal pleiotropy was observed (Tables S2 and S4). Steiger filtering further validated the directional relationship between gene expression and disease state (Table S5). Moreover, SERPINH1 and TMEM132C showed consistent directional effects across all four methods (Figure 3). Although PLTP emerged in discovery, it was excluded after validation showed an opposite effect direction.

**Figure 4:**
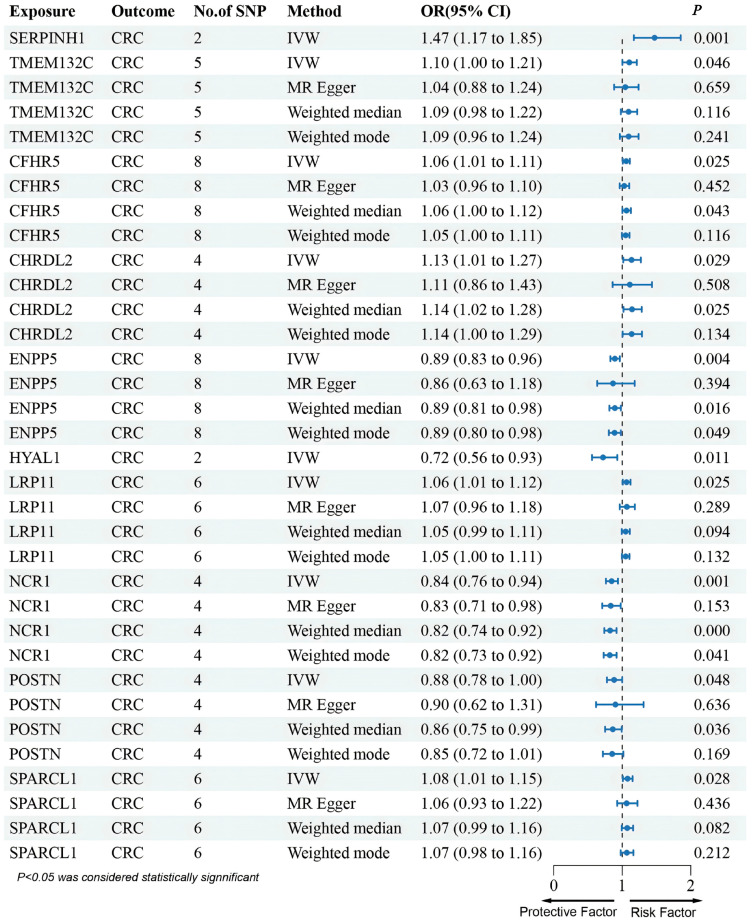
Forest plots illustrating the results of the validation stage for essential cis-pQTLs in deCODE.

Furthermore, although several genes were not found in the eQTLGen dataset, we conducted an MR analysis using cis-pQTL protein data from the UKB-PPP and deCODE databases. This Mendelian randomization analysis identified that CFHR5, CHRDL2, LRP11, and SPARCL1 were associated with an increased risk of CRC, whereas ENPP5, HYAL1, NCR1, and POSTN were linked to a decreased risk of CRC (Figure 3 and Figure 4).

### 3.3. Clinical Expression and Survival Analysis

**Figure 5:**
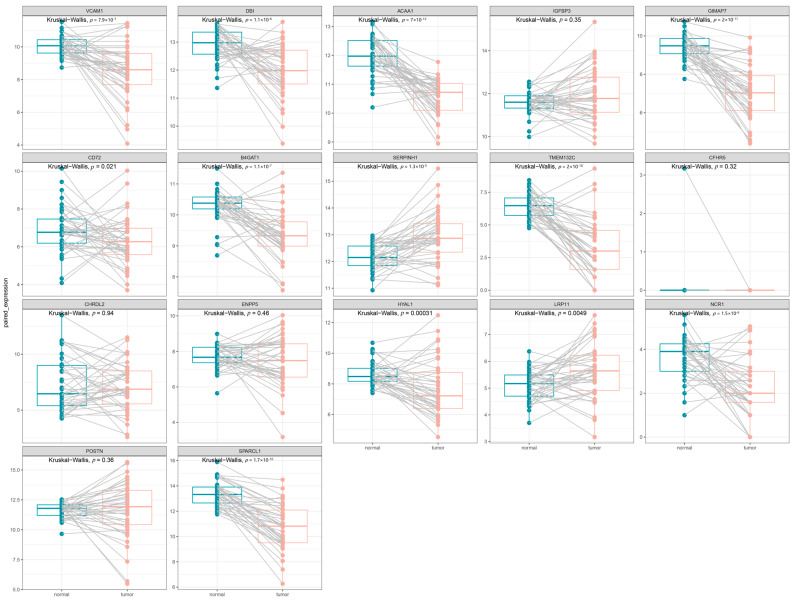
The expression of 17 candidate genes between the tumor and paired-normal tissues in CRC patients. Each panel displays the results of a Kruskal–Wallis test, with the corresponding *p*-value indicated at the top.

Using RNA-sequencing data from TCGA for CRC, we validated the previously identified candidate drug-associated genes. Figure 5 shows the significant differential expression between the tumor and paired-normal tissues. Specifically, *VCAM1* (*p* = 7.9 × 10^−7^), *DBI* (*p* = 1.1 × 10^−6^), *ACAA1* (*p* = 7.0 × 10^−12^), *GIMAP7* (*p* = 2.0 × 10^−11^), *CD72* (*p* = 0.021), *B4GAT1* (*p* = 1.1 × 10^−11^), TMEM132C (*p* = 2.0 × 10^−10^), *HYAL1* (*p* = 3.1 × 10^−4^), *NCR1* (*p* = 1.5 × 10^−6^), and *SPARCL1* (*p* = 1.7 × 10^−10^)were significantly overexpressed in tumor tissue, whereas *SERPINH1* (*p* = 1.3 × 10^−5^) and *LRP11* (*p* = 4.9 × 10^−3^)were significantly downregulated.

**Figure 6:**
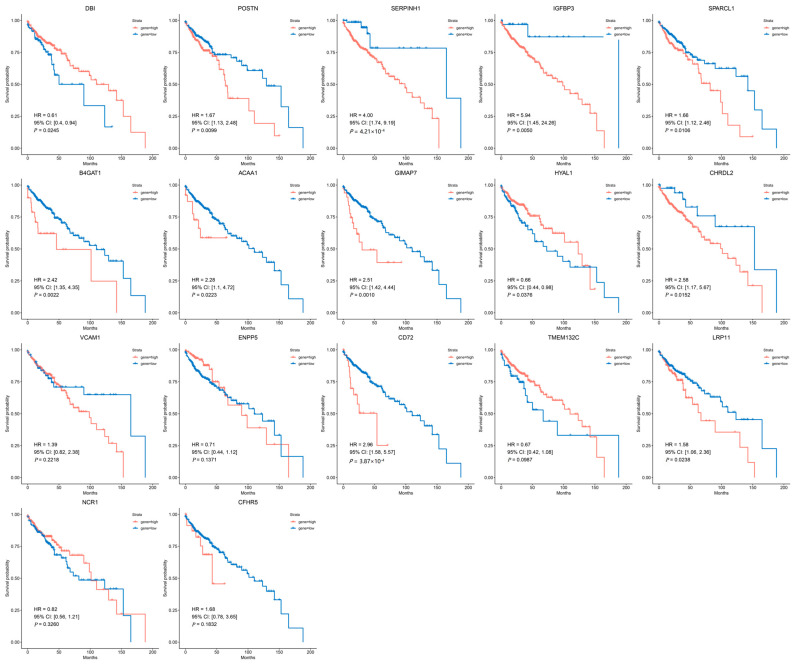
The survival analysis of 17 candidate genes in CRC patients. Kaplan–Meier survival curves for patients stratified by high (red line) or low (blue line) expression levels of each candidate gene.

Survival analysis indicated that *POSTN* (HR (95% CI) = 1.67 (1.13–2.48), *p* = 9.9 × 10^−3^), *SERPINH1* (HR (95% CI) = 4.00 (1.74–9.19), *p* = 4.21 × 10^−4^), *IGFBP3* (HR (95% CI) = 5.94 (1.45–24.26), *p* = 5.0 × 10^−3^), *SPARCL1* (HR (95% CI) = 1.66 (1.12–2.46), *p* = 1.1 × 10^−2^), *B4GAT1* (HR (95% CI) = 2.42 (1.35–4.35), *p* = 2.2 × 10^−3^), *ACAA1* (HR (95% CI) = 2.28 (1.10–4.72), *p* = 2.2 × 10^−2^), *GIMAP7* (HR (95% CI) = 2.51 (1.42–4.44), *p* = 1.0 × 10^−3^), *CHRDL2* (HR (95% CI) = 2.58 (1.17–5.67), *p* = 1.5 × 10^−2^), *CD72* (HR (95% CI) = 2.96 (1.58–5.57), *p* = 3.9 × 10^−4^) and *LRP11* (HR (95% CI) = 1.58 (1.06–2.36), *p* = 2.4 × 10^−2^) correlated with poorer overall survival (OS), whereas *DBI* (HR (95% CI) = 0.61 (0.40–0.94), *p* = 2.5 × 10^−2^) and *HYAL1* (HR (95% CI) = 0.66 (0.44–0.98), *p* = 3.8 × 10^−2^) were associated with improved OS (Figure 6).

However, except for *DBI*, *IGFBP3*, *CD72*, *SERPINH1*, *CHRDL2*, *HYAL1*, *LRP11*, and *SPARCL1*, the remaining nine genes were excluded due to inconsistent effect directions or lack of prognostic significance.

### 3.4. Immune Microenvironment Analysis of Candidate Genes in CRC

In this study, we employed ssGSEA analysis to quantify 28 immune cell infiltrates in TCGA CRC tumors. This method enables the quantification of the relative abundance of immune cells within each tumor sample, providing a comprehensive view of the tumor immune microenvironment (TIME). We correlated the expression of eight genes (*CD72*, *CHRDL2*, *DBI*, *HYAL1*, *IGFBP3*, *LRP11*, *SERPINH1*, and *SPARCL1*) with immune cell infiltration to elucidate their roles in the CRC TIME.

**Figure 7:**
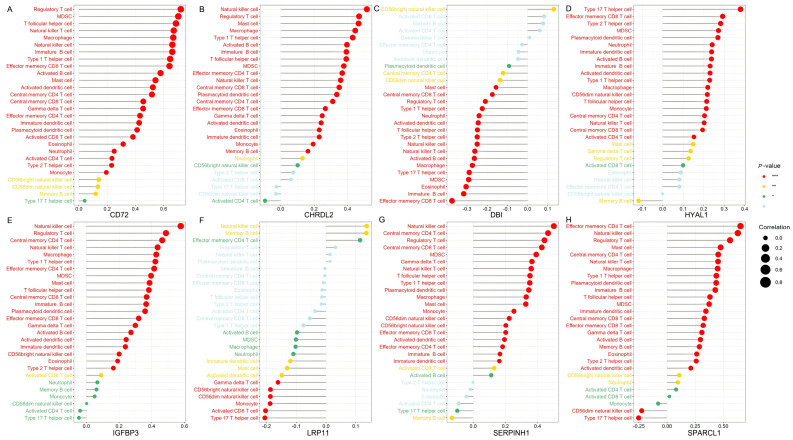
The immune microenvironment analysis of candidate genes in CRC. The ssGSEA analysis was conducted using CRC tumor expression data from TCGA database. Each panel illustrates Spearman correlation coefficients between the expression of a specific candidate gene and 28 immune cell types within CRC tissues. Panels depict results for candidate genes: (**A**) *CD72*, (**B**) *CHRDL2*, (**C**) *DBI*, (**D**) *HYAL1*, (**E**) *IGFBP3*, (**F**) *LRP11*, (**G**) *SERPINH1*, and (**H**) *SPARCL1*. The correlation strength is indicated by dot size, while significance levels are represented by dot color, with red indicating high significance (lowest *p*-value), transitioning through yellow and green, to grey for non-significant associations. Bonferroni correction was applied for multiple comparisons in the ssGSEA analysis involving 8 genes, and a threshold of *p* < 0.00625 (0.05/8) was considered statistically significant (*** *p* < 0.000125, ** *p* < 0.00125, * *p* < 0.00625).

*CD72* demonstrated a significant positive correlation with both immune-suppressive cells, such as regulatory T cells (Tregs) and myeloid-derived suppressor cells (MDSCs), and immune-activating cells, including CD8^+^ T cells and natural killer (NK) cells. This suggests that *CD72* may play a dual role in the immune microenvironment of CRC (Figure 7A).

*CHRDL2* shows a prominent positive correlation with key immune cell populations including NK cells, CD8^+^ T cells, Tregs, and MDSCs. The positive association with NK cells and CD8^+^ T cells suggests that *CHRDL2* may enhance cytotoxic immune responses, potentially supporting anti-tumor activity. Conversely, its correlation with Tregs and MDSCs, which are well-known mediators of immune suppression, implies that *CHRDL2* might also contribute to establishing an immunosuppressive microenvironment (Figure 7B).

*DBI* shows a negative correlation with Tregs and MDSCs, both of which are known to promote immune suppression in the TME. This suggests that *DBI* expression may be associated with a less immunosuppressive environment. Additionally, *DBI* exhibited a weak positive correlation with activated CD8^+^ T cells and activated CD4^+^ T cells, both of which are critical for anti-tumor immunity. This weak positive association implies that *DBI* may contribute to immune activation (Figure 7C).

*HYAL1* is positively correlated with effector memory CD8^+^ T cells and Type 17 helper (Th17) cells—both associated with anti-tumor immune responses—suggesting that *HYAL1* may contribute to the activation or maintenance of tumor-reactive immunity. On the other hand, its positive association with MDSCs, which are known for their immunosuppressive functions, indicates that *HYAL1* may also facilitate an immunosuppressive microenvironment (Figure 7D).

*IGFBP3* shows strong positive correlations with NK cells and weak correlations with memory B cells and Th cells, suggesting that it may enhance innate immunity, particularly NK cell function (Figure 7E).

*LRP11* shows a weak positive correlation with NK cells and negative correlations with both CD8^+^ T cells and Th17 cells, but its role in the tumor immune microenvironment (TME) of CRC appears to be more complex, potentially skewed toward immune suppression rather than activation (Figure 7F).

*SERPINH1* was associated with various immune cells, including NK cells, CD8^+^ T cells, Tregs and MDSCs. The dual associations of *SERPINH1* with both immune-activating cells (NK and CD8^+^ T cells) and immune-suppressive cells (Tregs and MDSCs) suggest that *SERPINH1* may play a complex role in the TME. It could act as a modulator, balancing immune activation and suppression to maintain TME homeostasis (Figure 7G).

*SPARCL1* is positively correlated with effector memory CD4^+^ T cells and NK cells, indicating a potential role in promoting both adaptive immunity and innate immune responses. Its correlation with MDSCs and Tregs suggests a dual role, where it may facilitate both immune activation and suppression, depending on the context of the tumor microenvironment (Figure 7H).

The eight candidate genes—*CD72*, *CHRDL2*, *DBI*, *HYAL1*, *IGFBP3*, *LRP11*, *SERPINH1*, and *SPARCL1*—are involved in regulating the tumor immune microenvironment in CRC by modulating various immune cell types. Genes like *DBI* are positively associated with immune-activating cells (CD8^+^ T cells) and negatively associated with immune-suppressive cells (Tregs and MDSCs), suggesting that they may enhance anti-tumor immunity. Some genes, such as *CHRDL2* and *HYAL1*, appear to play a dual role in TME by promoting the infiltration of Tregs and MDSCs, as well as immune-activating cells. On the other hand, understanding the complex roles of these genes can offer insights into potential therapeutic strategies aimed at modulating the immune landscape in CRC, either by promoting immune activation or overcoming immune suppression.

### 3.5. Analysis of Single-Cell Gene Expressions in CRC

**Figure 8:**
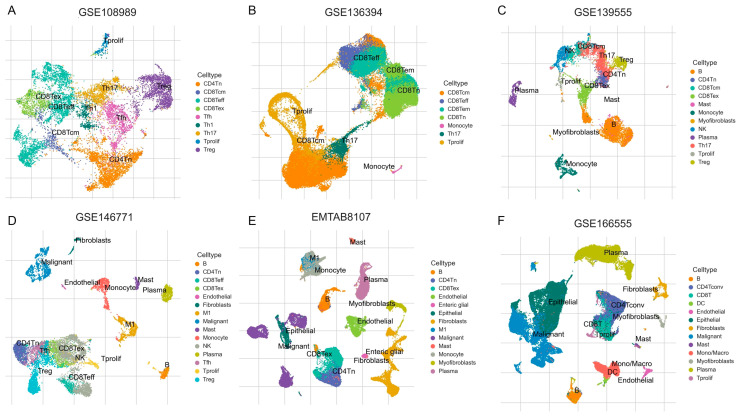
The t-SNE plots of scRNA-seq data from six independent CRC datasets: (**A**): GSE108989, (**B**): GSE136394, (**C**): GSE139555, (**D**): GSE146771, (**E**): EMTAB8107, and (**F**): GSE166555. Distinct colors represent different annotated cell types, including various T cell subsets (like CD4Tn, CD8Tem, Th17, Treg), B cells, natural killer (NK) cells, monocytes, macrophages, mast cells, fibroblasts, endothelial cells, myofibroblasts, plasma cells, dendritic cells (DC), proliferating cells (Tprolif), and malignant cells.

We assessed the expression of eight candidate genes—*CD72*, *CHRDL2*, *HYAL1*, *IGFBP3*, *LRP11*, *SERPINH1*, *SPARCL1*—across six scRNA-seq CRC datasets (GSE108989, GSE136394, GSE139555, GSE146771, EMTAB8107, and GSE166555). The gene expression was visualized in t-SNE plots for each dataset (Figure 8), and the expression levels of these genes across various cell types were quantified in heatmaps.

**Figure 9:**
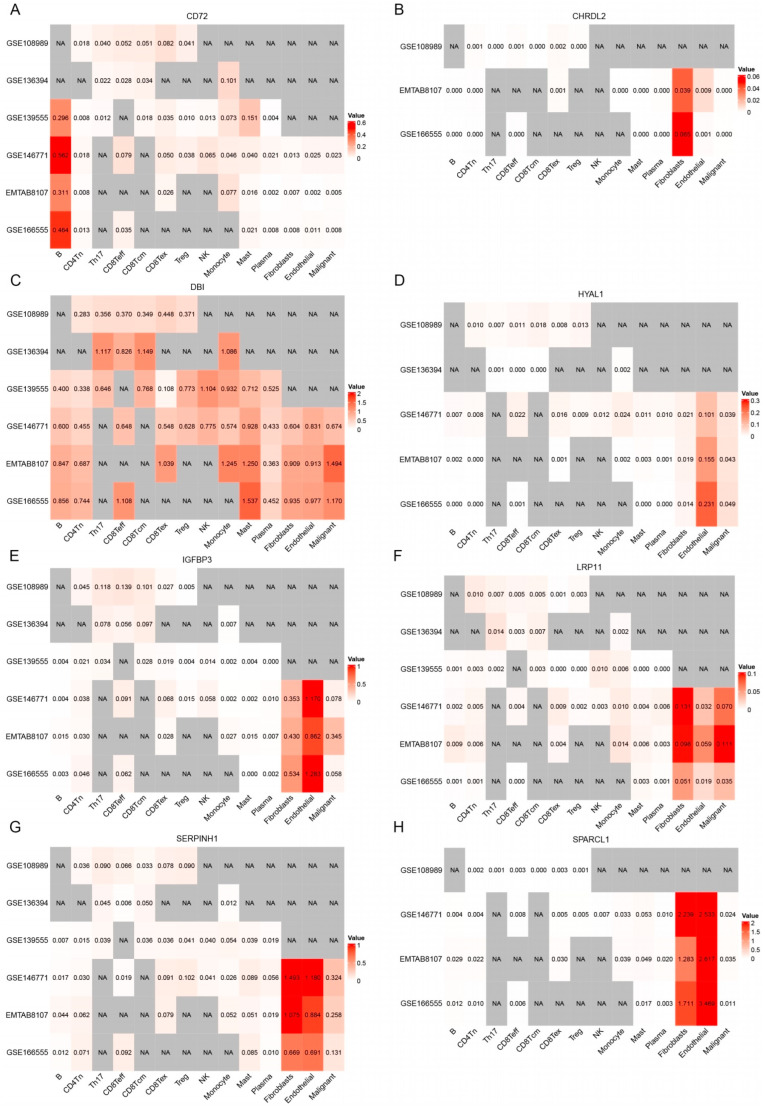
The expression patterns of eight genes (**A**–**H**) (*CD72*, *CHRDL2*, *DBI*, *HYAL1*, *IGFBP3*, *LRP11*, *SERPINH1*, and *SPARCL1*) across different single-cell populations identified in CRC datasets. The intensity of red color corresponds to higher gene expression levels, while white indicates low expression. Grey areas labeled “NA” represent cells where expression data were not available or detected.

*CD72* exhibited generally high expression in several immune cell types, including B cells, CD8Teff, and monocytes (Figure 9A). Additionally, *CD72* showed high expression in CD8Tex, suggesting its potential role in T cell exhaustion and immune modulation. A slight increase in *CD72* expression was also observed in Treg cells, indicating that *CD72* may play a role in regulating immune tolerance within TME. While the overall expression of *CD72* is not extremely high in all cell types, it appears to play a crucial role in enhancing B cell immunity, as well as a dual role in T cell-mediated immunity.

*CHRDL2* exhibited overall low expression levels across the dataset (Figure 9B). However, its expression was elevated in endothelial cells and fibroblasts, suggesting that it may have a more localized role in these specific cell types. Despite this increase in expression within endothelial cells and fibroblasts, the overall low expression of *CHRDL2* implies that its impact on the broader TME is likely to be modest.

*DBI* shows relatively higher expression in CD8Teff and CD8Tcm cells than in Tregs. This distribution suggests that *DBI* may be associated with T cell-mediated anti-tumor immunity, possibly supporting cytotoxic activity or memory responses against tumor cells (Figure 9C). *DBI* was also highly expressed in mast cells, implicating a potential role in inflammatory regulation within the TME.

*HYAL1* exhibited overall low expression levels, with its expression notably elevated in endothelial cells and malignant cells (Figure 9D). This suggests that while the gene is not highly expressed across all cell types, it may play a more localized role in TME, suggests that it could contribute to tumor progression by promoting angiogenesis and facilitating ECM remodeling, thus supporting tumor growth and metastasis.

*LRP11* exhibited overall low expression levels, but its expression was elevated in fibroblast, endothelial, and malignant cells (Figure 9F). The elevated expression of *LRP11* in fibroblast, endothelial, and malignant cells suggests a potential role in tumor development and progression, possibly through interactions with the ECM or influencing tumor cell migration and invasion.

*IGFBP3*, *SERPINH1*, and *SPARCL1* all exhibited higher overall expression levels, particularly in fibroblast and endothelial cells (Figure 9E,G,H). These cell types are key components of TME and are involved in processes such as ECM remodeling, fibrosis, and immune modulation.

### 3.6. Clinical Immunotherapy Response Validation

**Figure 10:**
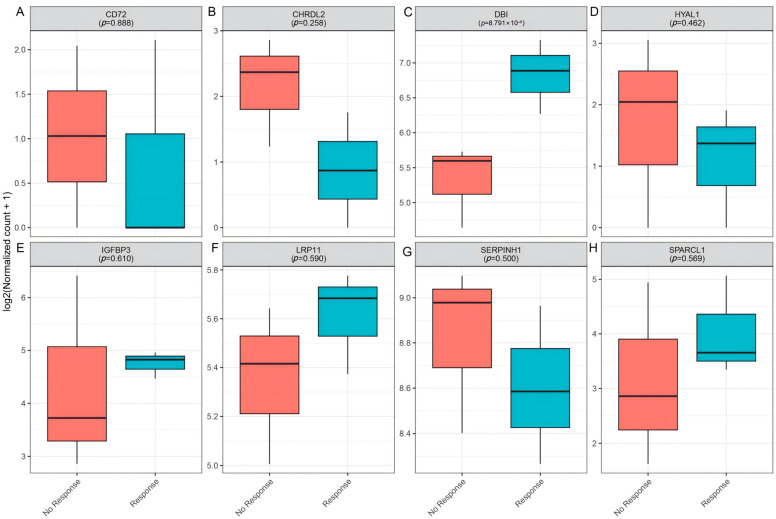
Validation of candidate genes in clinical immunotherapy response (GSE154538). (**A**–**H**): Boxplots depicting the expression levels of *CD72* (**A**), *CHRDL2* (**B**), *DBI* (**C**), *HYAL1* (**D**), *IGFBP3* (**E**), *LRP11* (**F**), *SERPINK1* (**G**), and *SPARCL1* (**H**) in non-responders (red) versus responders (blue).

To further investigate the role of candidate genes in mediating immunotherapy response, we analyzed their expression in GSE154538, a dataset consisting of advanced CRC patients with high TMB who were treated with the anti-PD-1 monoclonal antibody (nivolumab). Notably, *DBI* expression was significantly higher in responders (*p* = 8.79 × 10^−4^, Figure 10C), suggesting its association with favorable anti-PD-1 outcomes. In contrast, other candidate genes (*CD72*, *CHRDL2*, *HYAL1*, *IGFBP3*, *LRP11*, *SERPINH1*, and *SPARCL1*) showed no significant differences in expression between response groups (Figure 10).

### 3.7. Drug Target Prediction of DBI

An analysis of the DrugBank and TTD databases revealed that Coenzyme A is an approved DBI ligand, whereas hexadecanal represents an experimental DBI modulator. Meanwhile, using the DSigDB drug database on Enrichr, Benzo[b]fluoranthene, Dibenz[a,h]anthracene, baclofen, diltiazem, paclitaxel, ambroxol, puromycin, hydralazine and Vitinoin were the candidate drugs associated with DBI (Table 1).

## 4. Discussion

In this study, we integrated data from eQTLGen, UKB-PPP, and deCODE to identify genes significantly associated with CRC risk. This was followed by clinical expression and survival analyses, which validated eight genes (*IGFBP3*, *CD72*, *SERPINH1*, *CHRDL2*, *LRP11*, *SPARCL1*, *DBI* and *HYAL1*) as significant CRC candidates. *IGFBP3*, *CD72*, *SERPINH1*, *CHRDL2*, *LRP11*, and *SPARCL1* were linked to increased CRC risk, whereas *DBI* and *HYAL1* were associated with decreased risk.

Another key finding is the varied roles of these genes in the tumor immune microenvironment (TIME). *CD72*, *CHRDL2*, *SERPINH1*, *SPARCL1*, and *HYAL1*, identified as risk-associated or protective genes in CRC, exhibit dual roles in immune regulation within the TME. Their associations with both immune-activating (NK and CD8^+^ T cells) and immune-suppressive (Tregs and MDSCs) populations suggest they contribute to a complex balance between anti-tumor immunity and immune evasion. One plausible mechanism that may account for this observed duality involves ECM remodeling and angiogenesis, both of which contribute to shaping the tumor microenvironment by altering its physical and biochemical properties. These changes can influence immune cell infiltration, distribution, and activation. Specifically, ECM-associated genes such as *SERPINH1*, *SPARCL1*, and *HYAL1* have been implicated in modulating immune cell behavior within the tumor milieu. For instance, SPARCL1 has been shown to enhance vascular stability in CRC, a factor that may facilitate the infiltration of cytotoxic lymphocytes, including NK cells and CD8⁺ T cells [46]. Pan-cancer analyses reveal that SPARCL1 is associated with both pro-tumorigenic and anti-tumorigenic immune components. In several gastrointestinal malignancies, elevated SPARCL1 expression correlates with increased signatures of M2-polarized macrophages, suggesting a context-dependent immunosuppressive function [47]. These findings underscore the complex and potentially paradoxical role of SPARCL1 in modulating the tumor immune microenvironment. Another potential mechanism underlying the dual immunomodulatory roles of these genes may involve their interactions with various immune cell populations and key immunoregulatory factors—such as cytokines and immune checkpoint molecules—through which they can orchestrate both immunostimulatory and immunosuppressive responses. One study showed that soluble CD72 upregulates IL-17A and IFN-γ expression in activated CD4⁺ T cells and enhances their proliferation [48]. Conversely, another study found that high CD72 expression was associated with poor prognosis for kidney renal clear cell carcinoma (KIRC) patients and correlated positively with regulatory T cell infiltration and the expression of inhibitory molecules such as PD-1 and CTLA-4 [49]. In conclusion, further studies are needed to clarify how these genes regulate immune balance in the CRC.

In contrast, *IGFBP3*, *LRP11*, and *DBI* exhibit more distinct immune regulatory patterns. Although *IGFBP3* was identified as a risk-associated gene of CRC, its linkage to NK cells, memory B cells, and Th cells suggests a potential role in innate immunity and anti-tumor surveillance. Similarly, *LRP11*, a risk-associated gene, weakly associates with NK cells and negatively correlates with CD8^+^ T and Th17 cells, potentially promoting immune suppression via extracellular matrix (ECM) interactions in fibroblasts and endothelial cells. Notably, *DBI* was associated with an immune landscape potentially indicative of enhanced anti-tumor responses, characterized by inverse correlations with Tregs and MDSCs, alongside positive associations with activated CD8⁺ and CD4⁺ T cells.

DBI (also known as Acyl-CoA binding protein, ACBP) is an 87-amino-acid phylogenetically conserved protein. Intracellularly, it regulates oxidative metabolism and autophagy, whereas externally it is secreted via an autophagy-dependent pathway. Notably, when DBI is secreted into the extracellular space, it acts as a “tissue hormone” that inhibits autophagy in neighboring cells [50,51]. A study suggested that ACBP/DBI levels increase in various tissues, as well as in the plasma, in the context of aging, obesity, uncontrolled infection or cardiovascular, inflammatory, neurodegenerative, and malignant diseases in humans [52]. Protein expression profiling of ACBP/DBI confirmed that ACBP/DBI was strongly expressed by parenchymatous cells from specific human and mouse organs (e.g., kidney, large intestine, liver, lung, etc.) [53]. In addition, inhibition of ACBP/DBI has been demonstrated to mediate cardioprotective, hepatoprotective, and pneumoprotective effects [54]. Single-cell analyses in bladder cancer found DBI to be enriched in quiescent cancer stem cells and associated with higher tumor stemness and grade [55]. In the context of chemoimmunotherapy, DBI/ACBP neutralization may result in better cancer control in models of breast cancer, non-small cell lung cancer, and sarcoma [56]. However, it remains to be determined whether ACBP/DBI inhibition might affect the function of the gastrointestinal tract including esophagus, stomach, and colon, where this protein is highly expressed. Our findings indicated that higher *DBI* expression was associated with a reduced risk of colorectal cancer and improved patient survival, suggesting a potential tumor-suppressive role and prognostic relevance.

The influence of DBI on immune cell function may be intricately linked to its fundamental roles in cellular metabolism and autophagy. DBI/ACBP is a small intracellular protein with a high affinity for binding long-chain acyl-CoA molecules, thereby regulating lipid metabolism and promoting fatty acid oxidation (FAO) [57]. Importantly, immunosuppressive cell populations such as Tregs and MDSCs rely heavily on lipid-based metabolic pathways to sustain their suppressive functions, particularly within the nutrient-deprived conditions of TME. Tumor-infiltrating Tregs have been shown to preferentially engage FAO and oxidative phosphorylation (OXPHOS) over glycolysis for energy production [58], while MDSCs upregulate fatty acid uptake and β-oxidation to support their immunosuppressive activity [59]. Given DBI’s ability to sequester intracellular acyl-CoA, elevated DBI expression may disrupt lipid metabolic programs by altering free fatty acid availability and lipid signaling cascades. This disruption could impair the metabolic homeostasis required for the suppressive function of Tregs and MDSCs in the TME, thereby reducing immunosuppression and enhancing anti-tumor immune responses. In parallel, CD8⁺ T cells have been reported to benefit from enhanced FAO activity, which supports their persistence, functional fitness, and cytotoxic capacity in metabolically constrained environments [53]. Therefore, elevated DBI expression may also enhance CD8⁺ T cell fatty acid oxidation, supporting their persistence, effector function, and anti-tumor activity under nutrient limitation [60]. Collectively, these findings suggest that DBI may act as a metabolic regulator that differentially modulates immune cell function within the TME, with potential implications for metabolic reprogramming strategies to boost anti-tumor immunity.

CRC treatment has advanced significantly in recent years, yet challenges remain in improving patient outcomes, particularly in metastatic and treatment-resistant cases. The emergence of immunotherapy has transformed the management of CRC, particularly in patients with microsatellite instability-high (MSI-H) or mismatch repair-deficient (dMMR) tumors, where PD-1 inhibitors such as pembrolizumab and nivolumab demonstrate durable responses. However, for the majority of CRC patients with microsatellite stable (MSS) tumors, immunotherapy remains largely ineffective, highlighting the urgent need for novel combination strategies [61,62]. In our study, *DBI* expression in tumors was associated with an immune-active phenotype, with observed trends suggesting increased cytotoxic CD8⁺ T cell infiltration and lower regulatory Treg signatures in high-*DBI* tumors. These findings indicated that DBI may influence the immune microenvironment, potentially shifting the balance away from Treg-mediated immunosuppression and facilitating more effective CD8⁺ T cell anti-tumor responses. In recent years, T cell-based immunotherapy has yielded promising responses in some patients, particularly in those whose cancers have high MSI or deficient mismatch repair [63,64,65]. Therefore, DBI could potentially enhance the efficacy of immune checkpoint therapies.

Our integrated analytical framework, which combines eQTL data from the eQTLGen Consortium with pQTL data from the UKB-PPP and deCODE databases, offers several notable strengths. First, the multi-omics approach enhances the robustness of our findings by validating candidate druggable genes at both the transcriptomic and proteomic levels. The use of SMR and HEIDI tests allows us to distinguish pleiotropy from genetic linkage, thereby providing strong causal inference. Additionally, integrating clinical expression data, survival analysis, and immune microenvironment assessments from TCGA and single-cell RNA-sequencing data contributes to a comprehensive understanding of gene function in CRC.

However, some limitations must be acknowledged. Our reliance on European-derived datasets (eQTLGen, UKB-PPP, deCODE, and FinnGen) may limit the generalizability of our results to other populations and introduce potential population-specific biases. Moreover, while SMR and HEIDI tests are powerful tools, they are dependent on the quality of the underlying GWAS and eQTL data, and residual pleiotropy may still affect causal estimates. Finally, while this study provides valuable insights through comprehensive computational analyses, it is important to acknowledge the lack of direct experimental validation. A key limitation of our study is the absence of in vitro or in vivo validation of the identified gene targets and predicted drug candidates. Although our findings have been supported by multiple layers of genomic data, they remain associative in nature. Further experimental investigations are needed to establish the causal roles of these genes in colorectal cancer and to evaluate the efficacy of the predicted therapeutic agents. For instance, RT-qPCR and Western blotting can be used to validate DBI expression in relevant cell types or tissue samples. Moreover, CRISPR knockdown or RNA interference can provide insights into the functional role of DBI in T cell behavior. IHC can further elucidate spatial expression patterns and potential interactions within the immune microenvironment. Despite these limitations, the multifaceted approach employed here provides a strong foundation for identifying and validating potential therapeutic targets in CRC.

## 5. Conclusions

Our study identified eight candidate genes—*CD72*, *CHRDL2*, *DBI*, *HYAL1*, *IGFBP3*, *LRP11*, *SERPINH1*, and *SPARCL1*—that were significantly associated with CRC risk. These genes demonstrated consistent associations across the discovery, validation, and clinical expression analyses. Notably, *DBI* emerged as a potential protective factor, showing strong associations with improved patient survival, enhanced immune activation, and increased responsiveness to immunotherapy. The remaining proteins demonstrated diverse and complex functions within the tumor immune microenvironment. These findings offer valuable insights into CRC pathogenesis and underscore novel targets for precision diagnostics and immunotherapy.

## Figures and Tables

**Table 1:** Candidate drugs for DBI.

Term	Status	*p*-Value	Adjusted *p*-Value	Odds Ratio
Coenzyme A	Approved	N.A.	N.A.	N.A.
Hexadecanal	Experimental	N.A.	N.A.	N.A.
Benzo[b]fluoranthene	Candidate	3.65 × 10^−3^	4.29 × 10^−2^	19,927
Dibenz[a,h]anthracene	Candidate	3.90 × 10^−3^	4.29 × 10^−2^	19,922
baclofen	Candidate	1.17 × 10^−2^	8.58 × 10^−2^	19,766
diltiazem	Candidate	1.82 × 10^−2^	9.17 × 10^−2^	19,636
paclitaxel	Candidate	2.08 × 10^−2^	9.17 × 10^−2^	19,583
ambroxol	Candidate	3.11 × 10^−2^	9.53 × 10^−2^	19,378
puromycin	Candidate	3.61 × 10^−2^	9.53 × 10^−2^	19,278
hydralazine	Candidate	3.88 × 10^−2^	9.53 × 10^−2^	19,224
Vitinoin	Candidate	3.90 × 10^−2^	9.53 × 10^−2^	19,220

## Data Availability

Data are provided within the manuscript or supplementary information files.

## Supplementary Materials

The following supporting information can be downloaded at: https://www.mdpi.com/article/10.3390/biomedicines13051115/s1. Table S1: The positive genetic variations of eQTLs utilized in the discovery phase; Table S2: The heterogeneity and pleiotropy analysis in the significance results of MR analysis of UKB-PPP cis-pQTLs and CRC; Table S3: The instrumental variables in MR analysis of UKB-PPP cis-pQTLs on CRC; Table S4: The heterogeneity and pleiotropy analysis in the significance results of MR analysis of deCODE cis-pQTLs and CRC; Table S5: The instrumental variables in MR analysis of deCODE cis-pQTLs on CRC.
